# Impact of the COVID-19 Pandemic on Vaccine-Preventable Diseases in Mexico: A Time Series Analysis (2014–2024)

**DOI:** 10.3390/epidemiologia7010026

**Published:** 2026-02-11

**Authors:** María Fernanda Hernández-Batres, Sofía Bernal-Silva, Georgina Cristina Delgado-Juárez, Andreu Comas-Garcia

**Affiliations:** 1 School of Medicine, Universidad Cuauhtémoc San Luis Potos, Dr. Salvador Nava Martínez 3291, Col. Viveros, San Luis Potosí 78290, Mexico; ferhdezb@gmail.com (M.F.H.-B.); ginna405@gmail.com (G.C.D.-J.); 2 Department of Microbiology, School of Medicine, Universidad Autónoma San Luis Potosí, Av. Venustiano Carranza 2405, Lomas Los Filtros, San Luis Potosí 78210, Mexico; sofia.bernal@uaslp.mx

**Keywords:** COVID-19 pandemic, vaccine-preventable diseases, morbidity, mortality, vaccines

## Abstract

The COVID-19 pandemic has significantly impacted public health in Mexico. Background/Objectives: This study evaluated its impact on the frequency of vaccine-preventable diseases (VPDs) from 2020 to 2024. Methods: The analyzed information was extracted from the weekly epidemiological bulletins, which compile the suspected, probable, and confirmed cases reported to the Ministry of Health. The epidemiological behavior of VPDs was analyzed with endemic channels based on 2014–2019 data. An endemic channel is a graphical tool that is used to plot a central tendency and its limits; with this tool we can detect the presence of an epidemic and quantify it. Between 2020 and 2024, VPDs presented variable patterns due to the pandemic. Results: Rotavirus cases exhibited an 81% negative deviation in 2020 and a final 47% negative deviation in comparison with the expected values from 2014–2019. Chickenpox declined by 91% in 2020, with a partial recovery in reports afterward. Hepatitis A and B declined initially, but hepatitis B surpassed pre-pandemic levels later. Mumps declined by 45% in 2020, with a partial recovery, remaining 35% below expected reports. Meningeal and pulmonary tuberculosis increased by 125% and 33%, respectively. Human Papilloma Virus (HPV) infection and mild cervical dysplasia showed negative deviations, with partial increases later. However, severe dysplasia and in situ cervical cancer reports exceeded expected levels. Conclusions: Overall, several VPDs showed negative deviations, which could increase the size of the susceptible population. In contrast, increases in tuberculosis and HPV infection present a major challenge for health systems, given their chronic and high treatment costs.

## 1. Introduction

Vaccine-preventable diseases (VPDs) are infectious diseases caused by bacteria or viruses for which there is an effective vaccine that can significantly reduce or prevent illness, complications, and death when given appropriately. In Mexico, case definitions of vaccine-preventable diseases (VPDs) rely on laboratory or clear clinical confirmation, and the vaccination scheme defines which vaccines are given free of charge at specific ages. The vaccination program in Mexico includes vaccines such as Bacillus Calmette-Guérin Vaccine (BCG) at birth, multiple doses of hexavalent/pentavalent (DTP-HepB-Hib-polio), rotavirus, and pneumococcal in the first year; measles-mumps-rubeolla vaccine (MMR) and varicella in the second year and preschool booster; plus Human papilloma virus (HPV) for adolescents and Td/Tdap and influenza for older age groups and risk populations. These diseases are considered notifiable in the country, and the epidemiological reports are publicly accessible and can be found on the website of the Dirección General de Epidemiología of the Ministry of Health [1,2,3]. There are some vaccines that are not designed to provide complete or immediate prevention (HPV and BCG, for example) [4]. However, they are considered effective due to their excellent protection against infection by the etiological pathogen, as well as against the development of disease complications (HPV and BCG, respectively). Despite that, cervical dysplasia, pulmonary tuberculosis, and extrapulmonary are complications of HPV and *M. tuberculosis* infection, respectively, and these vaccines could impact the disease burden [5,6].

For each VPD, there is a threshold at which vaccination prevents pathogen transmission (herd immunity). For example, for measles it is 92–95%, for pertussis 80–94%, for diphtheria 83–85%, for mumps 75–88%, and for influenza 30–75% [7]. There are multiple factors that can affect vaccination coverage, recently described across five domains: socioeconomic, health system, vaccine beliefs, cultural/social, and communication/information. However, vaccination coverage is not the only determinant of the epidemiological behavior of diseases. The emergence of a new pathogen could disturb the epidemiological behavior of infectious diseases, as happened with the 2009 H1N1pdm influenza virus pandemic, which directly caused a delay in the respiratory sincitial virus (RSV), parainfluenza, and adenovirus epidemics and indirectly impacted VPDs through the disruption of vaccination programs (coverage fell in some countries), hospital overcrowding, and non-pharmaceutical interventions (NPIs) (masks, quarantines) that temporarily reduced pertussis and measles transmission [8,9,10].

During the COVID-19 pandemic, due to the use of a sentinel surveillance system employed by the Mexican government, data published from the National Epidemiological Surveillance System (SINAVE) underestimated the reported disease burden, particularly COVID-19 cases [2]. These declines in increased susceptibility and the weakened epidemiological surveillance may have led to misinterpretation of the impact of reduced vaccination uptake [11,12].

In addition to the factors mentioned, the COVID-19 pandemic caused a significant decrease in worldwide vaccination coverage, with 105 of 183 planned campaigns postponed or canceled, resulting in 1.933 billion doses lost in over 50 countries. For instance, the first wave of COVID-19 in the UK caused a 20% drop, mostly in the second MMR dose and pneumococcal boosters [13]. These reductions were more pronounced among the socioeconomically disadvantaged, indicating that the pandemic intensified pre-existing health disparities [14].

In Mexico, the 2016 National Health and Nutrition Survey (ENSANUT) showed that 51.7% of children under 12 months and 53.9% of those 12–35 months had complete immunization. In 2021, coverage fell to 31.1% for kids aged 12 to 35 months, and in 2022, it fell to 26.6%. This decline in vaccination coverage and the weakened epidemiological surveillance may have led to misinterpretation of the impact of reduced vaccination uptake [11,12]. The Mexican Vaccination Observatory also said that in 2020, the number of children under 12 months who got vaccinated went up by 9.8%, but it went down to 6.4% in 2021. MMR vaccine coverage remained below 40%, suggesting the importance of immunization initiatives [15,16].

The aim of our study was to analyze the impact of the COVID-19 pandemic on vaccine-preventable diseases in Mexico from 2020 to 2024, focusing on meningeal tuberculosis, pulmonary tuberculosis, hepatitis A and B, whooping cough, mumps, rotavirus, HPV, and chickenpox.

## 2. Materials and Methods

### 2.1. Data Extraction

Data were collected from the Weekly Epidemiological Bulletin (WEB) of the Mexican Ministry of Health (MoH). The bulletin, which is published weekly by the Mexican government, includes information about the number of consultations and new cases reported based on their primary diagnosis codes during the epidemiological week, as well as year-to-date cumulative data for diseases under epidemiological surveillance. The bulletin contains only aggregated counts and states that no personal data were processed. This bulletin considers confirmed, suspected, or probable cases according to the surveillance system and allows for adjusting the figures once epidemiological study results and laboratory data are available to confirm or rule out cases. In Mexico, there is no public database about vaccination status or coverage. Data employed includes suspected, probable, and confirmed cases, which could introduce information bias. However, this bias would be homogeneous across all diseases, all weeks, and all years. On the other hand, the objective of this study is to ecologically evaluate the information routinely available to physicians and decision-makers. It is important to notice that in Mexico, there is no public database about vaccination status or coverage.

We used weekly case reports for each VPD in the bulletin to create epidemic channels, employing data from the period prior to the pandemic (2014–2019). We downloaded data from the PDF files of bulletins from January 2024 to March 2024 and created a database. Meningitis (G00–G03, except G00.1), tuberculosis (A15–A17), hepatitis A (B15), hepatitis B (B16), mumps (B26), rotavirus (A0.8), HPV (B97.7), mild to moderate cervical dysplasia (N87.0-N87.1), severe cervical dysplasia and in situ cervical cancer (N87.2, D06), and chickenpox (B01) were all included in the database. Although cervical dysplasias are a late consequence of HPV infection and their progression is influenced by multiple factors, this study considers these diseases preventable by vaccination, as dysplasia will not develop if infection does not occur.

The database from which we obtained the case information presents the study’s limitations. To reduce bias, we only included syndromic diagnoses or infectious diseases for which the vaccine is available in Mexico. Despite the project being an ecological study, we employed the STROBE checklist for cross-sectional studies to design the study, evaluate the results, and write the manuscript.

The analysis uses publicly available data from the Mexican Ministry of Health’s weekly epidemiological bulletin, reported in aggregated form (by sex, age group, and federal entity). Provided that no individual-level, identifiable, or re-identifiable data were accessed or linked, an Institutional Review Board (IRB) approval and informed consent are typically not required for this type of secondary analysis of publicly available, aggregated surveillance data.

### 2.2. Endemic Channel Design

With the weekly reports for each disease, we assemble the epidemiological curves between 2014 and 2024. Thereafter, we computed the 25th, 50th, and 75th percentiles for each week with the data from 2014 to 2019. Using this information, we constructed the endemic channels, which represented the expected epidemiological behavior for each disease. Once each endemic channel was constructed, we calculated the excess or deficit of reports and the percentage of change weekly, annually, and for the pandemic period (2020 to 2024), as we previously reported [1]. To consider the seasonality of the data, we compare a week of a certain year against this week for the endemic channel. Therefore, we compare the same time points across different years. A second strategy was to employ data for six years; we selected this length of time to avoid major epidemiological changes over time.

### 2.3. Statistical Analysis

After reporting the medians and percentiles, we analyzed all data to determine their normality using the Shapiro–Wilk test. We evaluated the statistical differences between the endemic channel (2014–2019) and the pandemic period (2020–2024) using the Mann–Whitney U test. A two-tailed *p*-value < 0.05 was considered indicative of statistical significance. To assess the temporal autocorrelation of the data, an autocorrelation function (ACF) analysis was performed. This analysis was applied to all time series, considering a 52-week period. The length of all the data series was 572 weeks. In all cases, the two-tailed *p*-value obtained was *p* < 0.0001; therefore, the data are not randomly distributed and exhibit a clustering pattern, specifically a seasonal pattern. The software employed was SPSS (IBM) version 30.0.

### 2.4. Universal Vaccination Program

In Mexico, the universal vaccination program is free and offers the following vaccines: BCG, hexavalent vaccine, *Haemophylus influenzae* type B, rotavirus, conjugated pneumococci, influenza, Tdap, MMR, meales-rubeola (MR), and HPV. More information is available at the following link: https://www.gob.mx/salud/articulos/esquema-de-vacunacion (accessed on 2 October 2025).

## 3. Results

### 3.1. Data Description

Of the total diseases included in the Weekly Epidemiological Bulletin of the MoH, only 10 of them had weekly information that would allow us to perform the analysis. In the present study, we decided to include dysplasias and cervical cancer, as they are the result of a vaccine-preventable infectious disease.

### 3.2. Rotavirus

**Figure 1:**
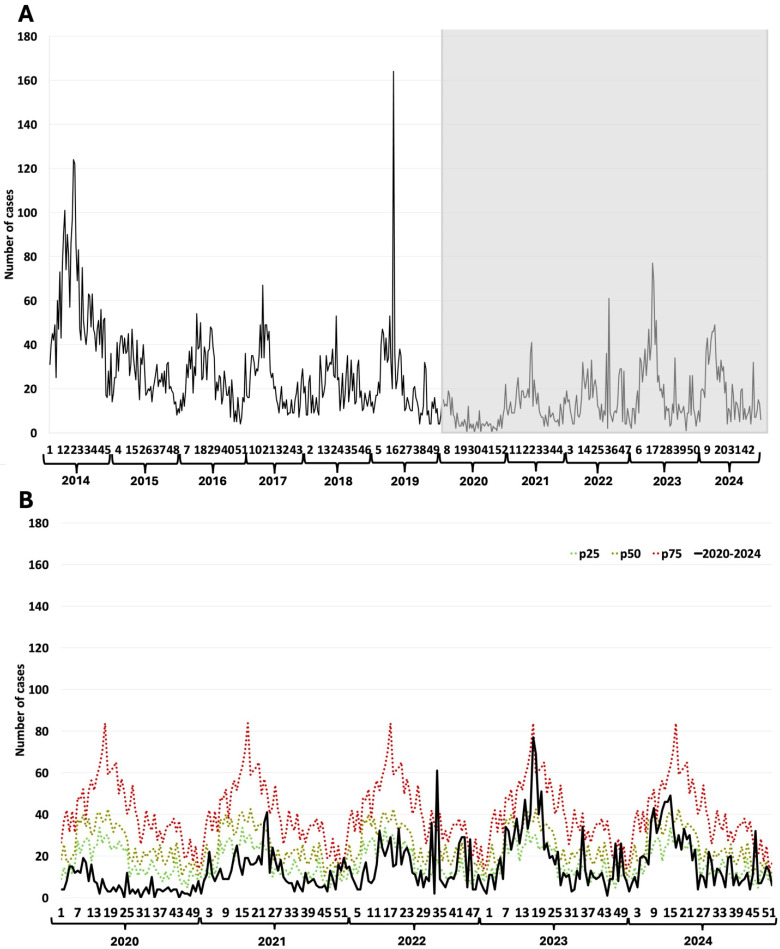
Rotavirus. (**A**) Weekly rotavirus detections between 2014–2024; (**B**) Weekly cases between 2020–2024 and endemic channel (2014–2024). The Y-axis represents the number of cases reported per epidemiological week. The X-axis represents the epidemiological weeks (1 to 52) for each year evaluated. The grayish rectangle indicates the COVID-19 pandemic period.

Typical periodic winter outbreaks characterize rotavirus patterns. These showed a breakdown during the first year of the COVID-19 pandemic, and the seasonal pattern was lost in subsequent years (Figure 1A). Towards the end of the studied pandemic period, rotavirus cases rose progressively, with a partial seasonal recovery (Figure 1A). Using the endemic channel showed that the weekly cases were lower than 25% of the usual levels during the first year of the pandemic. During 2021 and 2022, the number of cases remained below the 25th percentile. The number of cases was between the 25th and 50th percentiles by 2023; only in the last year did the curve regain its seasonal pattern while keeping a low number of cases (Figure 1B).

### 3.3. Meningeal and Pulmonary Tuberculosis

**Figure 2:**
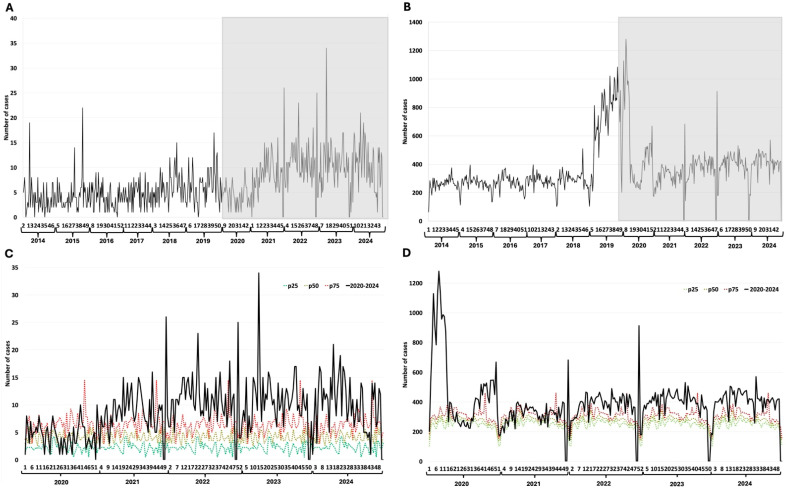
Tuberculosis. (**A**) Weekly detections between 2014–2024 of meningeal tuberculosis; (**B**) Weekly detections between 2014–2024 of pulmonary tuberculosis; (**C**) Weekly cases of meningeal tuberculosis between 2020–2024 and endemic channel (2014–2024); (**D**) Weekly cases of pulmonary tuberculosis between 2020–2024 and endemic channel (2014–2024). The Y-axis represents the number of cases reported per epidemiological week. The X-axis represents the epidemiological weeks (1 to 52) for each year evaluated. The grayish rectangle indicates the COVID-19 pandemic period.

Meningeal tuberculosis shows a constant trend, with a slight increase in case reporting towards the end of the prepandemic period. However, the increase in case reporting during the pandemic period is notable (Figure 2A). The same pattern is observed for pulmonary tuberculosis, except for the cases in 2019 and the beginning of 2020, a period that shows a very significant increase in the number of reported cases (Figure 2B). The increase in the number of reported cases remains above the 75th percentile of the endemic channel, with a trend toward a steady increase in the number of cases (Figure 2C). The study of how pulmonary tuberculosis cases changed over time shows a slow and steady rise during the pandemic, pushing the number of cases above the 75th percentile of the endemic channel, even though there were fluctuations in seasonal patterns during the first year of the pandemic (Figure 2D).

### 3.4. Chickenpox and Mumps

**Figure 3:**
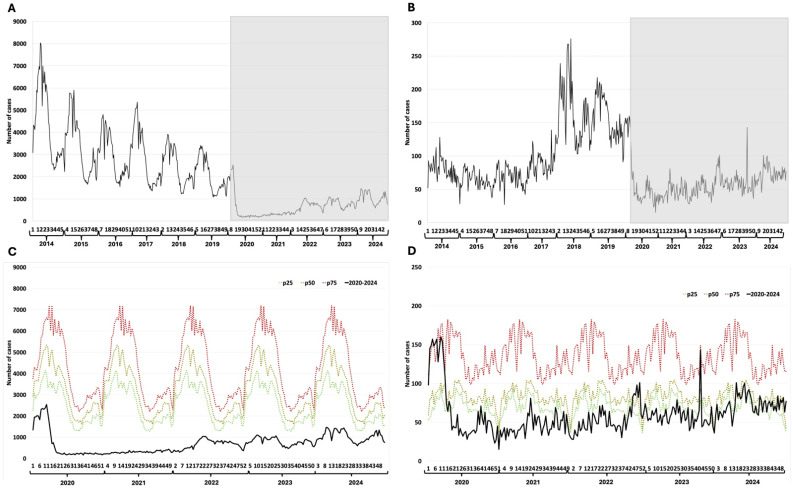
Chickenpox and mumps. (**A**) Weekly detections between 2014–2024 of chickenpox; (**B**) Weekly detections between 2014–2024 of mumps; (**C**) Weekly cases of chickenpox between 2020–2024 and endemic channel (2014–2024); (**D**) Weekly cases of mumps between 2020–2024 and endemic channel (2014–2024). The Y-axis represents the number of cases reported per epidemiological week. The X-axis represents the epidemiological weeks (1 to 52) for each year evaluated. The grayish rectangle indicates the COVID-19 pandemic period.

The endemic behavior of chickenpox had a characteristic seasonality with clear peaks at the beginning of each year, including the first year of the pandemic. The surveillance curve shows an increase in the number of reported cases after 2020, and seasonal peaks begin to be observed in the subsequent years (Figure 3A) and yet remain consistently below the epidemic lower threshold (Figure 3C). Mumps does not show seasonality. Reported cases increased from 2018 through the end of 2019. The decline in cases at the start of the pandemic is evident (Figure 3B). All reported cases of mumps during the pandemic period have remained below the 25th percentile of the epidemic channel (Figure 3D).

### 3.5. Hepatitis A and B

**Figure 4:**
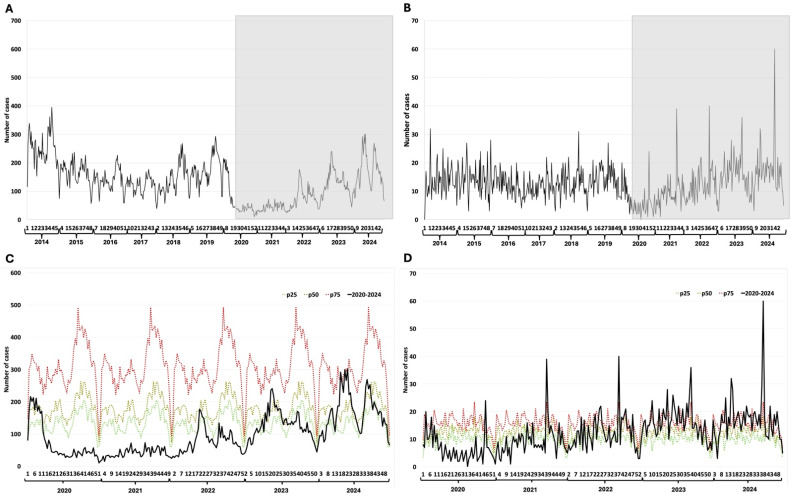
Viral hepatitis. (**A**) Weekly detections between 2014–2024 of hepatitis A; (**B**) Weekly detections between 2014–2024 of hepatitis B; (**C**) Weekly cases of hepatitis A between 2020–2024 and endemic channel (2014––024); (**D**) Weekly cases of hepatitis B between 2020–2024 and endemic channel (2014–2024). The Y-axis represents the number of cases reported per epidemiological week. The X-axis represents the epidemiological weeks (1 to 52) for each year evaluated. The grayish rectangle indicates the COVID-19 pandemic period.

Hepatitis A shows a decline in reported cases over time; however, since 2018, there has been an increasing trend in the number of cases (Figure 4A). During the first two years of the pandemic, the weekly cases were below the 25th percentile. For 2024, the weekly cases were between the 25th and 75th percentiles (Figure 4C). Hepatitis B cases decrease only during the first year of the pandemic (Figure 4B). Furthermore, an increase in the number of cases was observed in subsequent pandemic years, which quickly brought the curve back to levels expected according to the endemic curve (Figure 4D).

### 3.6. HPV and Cervical Dysplasia

**Figure 5:**
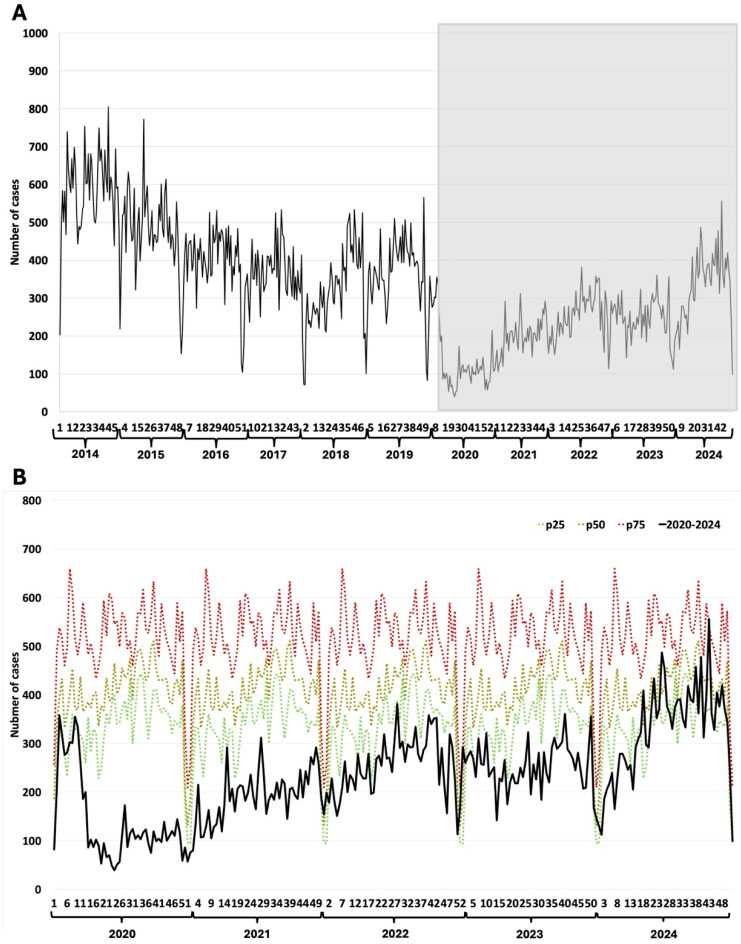
HPV detection. (A) Weekly HPV detections between 2014–2024; (**B**) Weekly cases between 2020–2024 and endemic channel (2014–2024). The Y-axis represents the number of cases reported per epidemiological week. The X-axis represents the epidemiological weeks (1 to 52) for each year evaluated. The grayish rectangle indicates the COVID-19 pandemic period.

The temporal pattern of HPV infection shows a decrease in cases with an upward trend in the two years prior to the pandemic. This epidemiologic pattern changed during the pandemic, showing a decrease in cases at the beginning with a gradual increase toward the end of the pandemic period studied (Figure 5A). During the pandemic period (2020–2024), a gradual increase in cases can be observed, barely reaching the 25th percentile in the last year of the study (Figure 5B).

**Figure 6:**
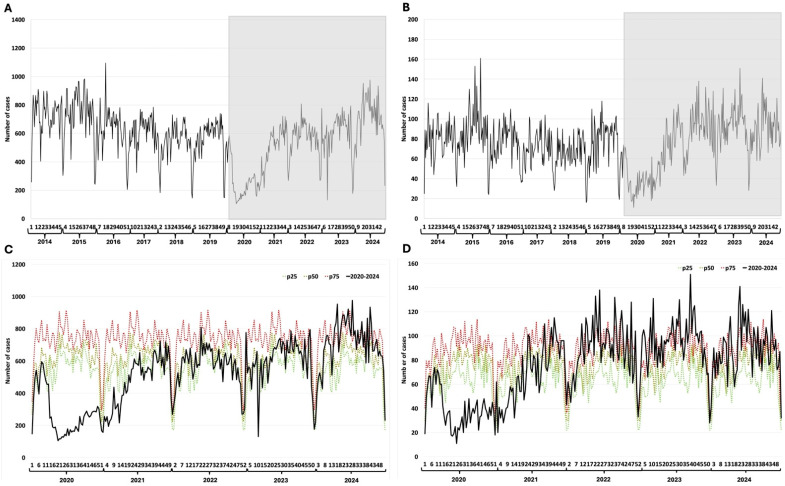
HPV detection. (**A**) Weekly detections between 2014–2024 of mild-moderate cervical dysplasia; (**B**) Weekly detections between 2014–2024 of severe cervical dysplasia and in situ cervical cancer; (**C**) Weekly cases of mild-moderate cervical dysplasia between 2020–2024 and endemic channel (2014–2024); (**D**) Weekly cases of severe cervical dysplasia and in situ cervical cancer; between 2020–2024 and endemic channel (2014–2024). The Y-axis represents the number of cases reported per epidemiological week. The X-axis represents the epidemiological weeks (1 to 52) for each year evaluated. The grayish rectangle indicates the COVID-19 pandemic period.

Mild-moderate dysplasia, severe dysplasia, and in situ cervical cancer are similar, with a downward trend that is interrupted during the first year of the pandemic with a subsequent increase in the number of reported cases (Figure 6A,B). After the first year of the pandemic, a clear increase was observed in all three grades of dysplasia and in situ cervical cancer. The increase was rapid and exceeded the 75th percentile by the end of the study period (Figure 6C,D).

### 3.7. Comparison Between Prepandemic vs. Pandemic Period

Medians were determined for the pre-pandemic period and for each year of the pandemic period. Percentage changes were negative for most diseases studied, except for meningeal and pulmonary tuberculosis, which showed a positive change during the pandemic (Supplementary Table S1).

The percentage change for other diseases started negative, and at different times throughout the pandemic period, it showed a positive change. Severe dysplasias and in situ cervical cancer showed this positive change from 77 to 87 weekly median cases (13%) for 2022; hepatitis B from 13 to 16 weekly median cases (19.2%) by 2023; and hepatitis A from 155 to 166 weekly median cases, as well as mild and moderate dysplasias from 643 to 708 weekly median cases for 2024 (7.1% and 10.2%, respectively). Rotavirus, chickenpox, mumps, and HPV infection consistently showed a negative percentage of change throughout the pandemic period.

During the excess or deficit case analysis, all diseases studied, except for pulmonary and meningeal tuberculosis, showed a deficit during the first two years of the pandemic (Table 1). Throughout the rest of the pandemic period, these diseases showed changes at different times. Severe dysplasia and in situ cervical cancer showed this change in 2022, in an excess of 722 cases. Hepatitis B showed a change during 2023, with an excess of 173 cases, and it was not until the last year of the study that mild and moderate dysplasia demonstrated a change with an excess of 4331 cases. Rotavirus always showed a deficit of cases throughout the pandemic period, presenting a deficit of 312 cases in 2024. Chickenpox, mumps, hepatitis A, and HPV infection also showed a deficit of cases during each year of the pandemic period.

Finally, comparing the pandemic vs. the prepandemic period (2014–2019 vs. 2020–2024), the average number of reported cases showed a significant difference (*p* < 0.00001) for the evaluated diseases, except for hepatitis B and severe dysplasia and in situ cervical cancer (Table 2).

After comparing the percentage of change between these two periods, we observe a reduction in the cases reported (negative percentage of change) for rotavirus, chickenpox, mumps, hepatitis A, HPV infection, and mild and moderate dysplasia. The percentage of change was positive for meningeal and pulmonary tuberculosis when comparing the periods.

## 4. Discussion

Despite not being a novel technique, the endemic channel is a valid epidemiological strategy used to visualize and analyze disease behavior over a period of time and in a specific region. Using this technique, we constructed epidemic curves with a central trend line and intervals defining safe, alert, and epidemic zones. This technique allows other researchers to evaluate their data and conduct a fair comparison. This paper aimed to avoid a highly precise quantification. From an ecological perspective, the analyzed information reveals changes in seasonality and disease patterns and provides a quantitative assessment of the behavior of VPDs. The first limitation of the study is that the cases evaluated include confirmed, suspected, or probable cases. A second limitation is that we do not know the sex, age, or vaccination status of the reported cases; therefore, we cannot perform a subanalysis considering these variables.

The first year’s results showed that the number of reported cases went down at first, but then slowly went up as restrictions were lifted. These patterns likely reflect the effectiveness of NPIs, as reduced mobility and reduced person-to-person contact modulated pathogen transmission. The initial pandemic results were consistent with global reports, with a decline in VPDs due to strict social distancing, mobility restrictions, and other strategies. A meta-analysis with global representation estimated that NPIs reduce transmission of SARS-CoV-2 by 49.5% after 2 weeks and 36.2% after 4 weeks. In China, NPIs in 2020 reduced the transmission of other diseases, for example, seasonal influenza by 89%, measles by 88%, scarlet fever by 86%, rubella by 75%, varicella by 43%, mumps by 36%, infectious diarrhea by 32%, and tuberculosis by 15%. In Mexico, each state government initiated NPIs over three weeks after the first confirmed case. Mexico’s response was slower and less centralized, resulting in significant variation in strictness between regions compared to other Latin American countries [10,17,18,19,20,21].

The reduction in the reports of VPD cases cannot be only explained by the NPIs. Other factors that could be considered are the viral interference and disruption of the surveillance systems, among others. Our research group has previously studied the factors mentioned. The phenomenon of viral interference (also known as viral superinfection) between H1N1 influenza and RSV was evaluated in Mexico, finding that circulation of the former suppressed the circulation of RSV. The viral interference has been reported as well in ecological and mathematical studies both in Mexico and in other countries (Stockholm and the USA). Regarding VPDs, we consider of interest the possible evaluation of viral interference, particularly in mumps and rotavirus [22,23,24,25].

Likewise, for the case of disruption of the surveillance systems due to the pandemic, we have previously estimated that the COVID-19 pandemic was linked to a decrease in case reports between 2020 and 2022, between 19.7% and 39.9% [1]. Similar underreporting occurred worldwide due to a lack of testing and political denial of COVID-19 during the pandemic. Other authors have postulated that underreporting infectious diseases is a challenge to public health that has emerged as a central issue in characterizing the dynamics of the COVID-19 pandemic. Underreporting is a complex phenomenon that is driven by factors specific to pathogens, country health systems, and politics [26,27,28].

Each of the diseases studied has a particular epidemiological behavior. On one hand, meningeal tuberculosis and hepatitis B have increased every year since 2014. The pattern suggests an endemic transmission without major seasonal fluctuations. During the pandemic, meningeal tuberculosis cases increased significantly; one explanation for this increase could be the reduction in vaccination coverage and campaigns. This reduction might explain why in Mexico the Tb cases increased during the pandemic, while in China the NPIs reduced the cases [11,12,15,29]. After the first year of the pandemic, hepatitis B had experienced a rapid increase, with cases almost reaching pre-pandemic levels. This finding aligns with previous studies that indicate an increase in cases and deaths due to interruptions in vaccination, particularly due to the slow transmission and the latency period [1,10,30].

These two diseases are preventable by vaccination, so the pattern is likely to change if coverage reaches between 44.1% and 70.6% for tuberculosis and between 79.6% and 85.7% for hepatitis B. The effectiveness of the BCG vaccine for meningeal tuberculosis is 80–85%, and the effectiveness of the hepatitis B vaccine is about 85–90%. The pandemic clearly increased cases of both diseases. Therefore, the low vaccination rate and vaccine effectiveness could explain the increase in these two diseases during the pandemic. This increase is relevant due to the high healthcare costs associated with these diseases, as well as the direct health impact. Therefore, it is crucial to direct public policy efforts to increase vaccination coverage [30,31,32,33,34].

On the other hand, a number of previously studied diseases demonstrated an epidemiological pattern, suggesting a potential decline in the number of cases. This scenario is the case with rotavirus, hepatitis A, chickenpox, and HPV. However, the pandemic altered their epidemiological behavior, and we now see an increasing number of cases. Mexico’s rotavirus detection pattern, similar to China’s, showed an initial drop in cases but remained low in 2020. Lappe et al.’s predictions about a resurgence could apply to the Mexican population, with cases not exceeding pre-pandemic levels by 2025. In Mexico, rotavirus vaccination has strict age limits, where the first dose must be administered before 6 months of age and the second dose before 8 months. This means that missed rotavirus vaccination cannot be caught up after these ages, leading to an accumulation of susceptible individuals in subsequent years [35,36].

Chickenpox showed the greatest reduction, consistent with previous studies on respiratory diseases due to NPIs. This supports Suzuki’s predictions of a decline in the post-pandemic period. Low vaccination rates and high susceptibility may lead to outbreaks in Mexico [29,37]. For instance, in Mexico, cervical screening decreased between 65% and 68% in the large public Mexican health system during the first nine months of the pandemic (Instituto Mexicano del Seguro Social) [38]. In this work, we observed that during the pandemic, HPV detection reports initially decreased but then subsequently increased. Due to a lack of early diagnosis (not necessarily related to the pandemic), we will now begin to detect an increase in advanced or complicated stages over the years since the start of the pandemic.

Improving vaccination uptake could mitigate the impact of diseases like rotavirus, chickenpox, and hepatitis A. However, HPV control will take longer (approximately 10 years, considering the pathophysiology of the disease). Meanwhile, the health impact and associated costs are perhaps inevitable, especially considering that reported cases of HPV were already increasing prior to the pandemic [9,21,29,39]. The above is consistent with previous studies indicating a decrease in disease reporting, low vaccination coverage, and increased cases and deaths due to vaccine interruptions during the pandemic (including HPV vaccination) [40].

Pulmonary tuberculosis and mumps each presented two significant waves of cases even before the pandemic began. This wave has occurred for pulmonary tuberculosis since 2018 and for mumps since 2019. This trend highlights the importance of establishing close epidemiological surveillance. A crucial aspect in the case of mumps that could be considered is the possible increase in the size of the susceptible population post-pandemic. It could have occurred for two possible reasons: the drastic reduction in the number of cases combined with the drop in vaccination rates. Therefore, unless vaccination campaigns are carried out, immunization schedules are restored, and early case detection is combined with sanitary cordons, the risk of large-scale outbreaks in the coming years should be taken into account. Changes in vaccination rates could directly influence morbidity and mortality, consistent with other studies that reported global drops in vaccination coverage followed by partial recovery [12,16,22,40]. The lack of reduction in pulmonary tuberculosis due to COVID-19 could suggest that the public health impact of this disease could be higher than that of other diseases reported. The reduction in vaccine coverage, which started in 2017, could also explain the excess of cases [11].

The pandemic’s impact on VPDs is clear in annual percentage changes between 2020 and 2024 and when we compare pre-pandemic vs. pandemic periods. A modeling study estimated that a 10% decrease in immunization will result in 11% of VPD-related deaths during the next decade. The WHO reported a 41% vaccination disruption in 2020 in 123 countries [41,42]. The impact of VPD-related deaths and vaccination disruption is directly relevant for diseases for which vaccination has an immediate protective effect. However, the epidemiological impact of other cases, such as the development of HPV lesions, will manifest over a longer period.

Finally, of interest are our findings, which suggest that the population is in an immunological state known as immune debt, a situation where control measures reduce exposure to pathogens by limiting person-to-person contact.

“Immune debt” is a concept that emerged due to the reduction of local pathogens during NPIs against COVID-19, which decreased population immunity by preventing natural exposure to respiratory and gastrointestinal pathogens. For example, a study conducted in Europe showed that there were reductions in respiratory infections followed by an increase. A study in Taiwan indicated that influenza hospitalizations fell by 90% during pandemic lockdowns and rebounded twentyfold when NPIs were lifted, but they did not exceed pre-pandemic levels thanks to ongoing vaccinations and voluntary NPIs.

This suggests that the greater the drop in infections, the larger the rebound that occurs afterwards. It is important to mention that these dynamics do not follow a linear pattern. Mathematical models have predicted an increase in varicella, rotavirus, and meningococcus cases if we do not increase the vaccination coverage [43,44,45,46].

## 5. Conclusions

The COVID-19 pandemic in Mexico caused significant changes in the report, patterns, transmission, and magnitude of all VPDs studied, potentially increasing the size of the susceptible population. Due to the potential immune debt, it would be advisable for the government to enhance vaccination catch-up strategies and early outbreak detection. It is important to notice that some diseases, such as meningeal tuberculosis, hepatitis, and associated HPV lesions, will require a longer period for the vaccine to take effect, potentially resulting in a high cost in the coming year.

## Figures and Tables

**Table 1:** Calculated deficit or excess of cases of vaccine-preventable diseases (VPD) reports in Mexico between 2020–2024.

Disease	Excess or Deficit (2020)	Excess or Deficit (2021)	Excess or Deficit (2022)	Excess or Deficit (2023)	Excess or Deficit (2024)
Rotavirus, median (p25–p75)	−993 (−1820 to −576)	−625 (−1452 to −208)	−520 (−1347 to −103)	−242 (−1069 to 175)	−312 (−1139 to 105)
Meningeal tuberculosis, median (p25–p75)	−10 (−108 to 343)	245 (127 to 343)	348 (230 to 446)	331 (213 to 446)	302 (184 to 400)
Pulmonary tuberculosis, median (p25–p75)	11,969 (10,099 to 13,322)	1889 (19 to 3242)	5471 (3601 to 6824)	5866 (3996 to 7219)	6573 (4703 to 6573)
Chickenpox, median (p25–p75)	−128,349 (−183,243 to −92,132)	−150,252 (−205,146 to −114,035)	−131,156 (−186,050 to −94,939)	−124,814 (−179,708 to −88,597)	−117,799 (−166,693 to −75,582)
Mumps, median (p25–p75)	−723 (−3496 to −2)	−1953 (−4.726 to −1.232)	−1514 (−4287 to −793)	−1268 (−4041 to −547)	−517 (−3.290 to 204)
Hepatitis A, median (p25–p75)	−5492 (−12,156 to −3174)	−7156 (−13.820 to −4838)	−5274 (−11,938 to −2956)	−2579 (−9243 to −261)	−531 (−7195 to 17,897)
Hepatitis B, median (p25–p75)	−314 (−483 to −165)	−172 (−341 to −23)	−18 (−187 to 132)	173 (4 to 323)	220 (51 to 370)
HPV, median (p25–p75)	−13,750 (−19,608 to −9966)	−11,054 (−16,912 to −7270)	−7678 (−13,536 to −3894)	−7772 (−13,630 to −7772)	−3711 (−9569 to 73)
Mild to moderate cervical dysplasia, median (p25–p75)	−18,043 (−24,082 to −13,973)	−7.622 (−13,661 to −3552)	−2366 (−8404 to 1705)	−927 (−6966 to 3144)	4331 (−1709 to 8401)
Severe cervical dysplasia and in situ cervical cancer, median (p25–p75)	−1867 (−2594 to −1227)	−316 (−1.043 to 324)	722 (−6 to 1361)	1019 (292 to 1658)	734 (7 to 1373)

Median estimation of weekly reports (p25–p75) obtained by comparing weekly cases with the endemic channel (2014–2019 period).

**Table 2:** Weekly accumulated median and excess or deficit of cases of VPD reports during the period 2020–2024 in Mexico.

Disease	Weekly Median Cases (2014–2019)	Weekly Median Cases (2020–2024)	Total Excess or Deficit (2020–2024)	% Change (2020–2024)
Rotavirus, median (p25–p75)	21 (15 to 37)	11 * (6 to 19)	−2692 (−6828 to −607)	−47.6%
Meningeal tuberculosis, median (p25–p75)	4 (3 to 7)	8 * (6 to 12)	1234 (645 to 1726)	125.0%
Pulmonary tuberculosis, median (p25–p75)	285 (255 to 320)	380 (301 to 435)	23,777 (3724 to 29,790)	33.2%
Chickenpox, median (p25–p75)	2767 (1909 to 2767)	681 * (296 to 923)	−646,368 (−920,841 to −465,283)	−75.4%
Mumps, median (p25–p75)	86 (68 to 133)	56 * (43 to 69)	−5975 (−19,840 to −2370)	−35.1%
Hepatitis A, median (p25–p75)	155 (120 to 199)	77 * (43 to 139)	−21,030 (−54,350 to −9442)	−50.6%
Hepatitis B, median (p25–p75)	13 (10 to 16)	11 (7 to 16)	−111 (−955 to 637)	−15.4%
HPV, median (p25–p75)	414 (343 to 493)	234 * (169 to 293)	−43,963 (−73,252 to −25,045)	−43.6%
Mild to moderate cervical dysplasia, median (p25–p75)	643 (557 to 725)	564 * (368 to 665)	−24,626 (−54,824 to −4275)	−12.3%
Severe cervical dysplasia and in situ cervical cancer, median (p25–p75)	77 (62 to 90)	79 (54 to 98)	292 (−3344 to 3487)	2.6%

Mann–Whitney U test compared to the endemic channel (2014–2019 period) * *p* < 0.00001.

## Data Availability

All data are available at https://www.gob.mx/salud/acciones-y-programas/historico-boletin-epidemiologico (accessed on 2 October 2025) and http://www.dgis.salud.gob.mx/contenidos/basesdedatos/da_defunciones_gobmx.html (accessed on 2 October 2025).

## Supplementary Materials

The following supporting information can be downloaded at https://www.mdpi.com/article/10.3390/epidemiologia7010026/s1, Table S1: Weekly median of vaccine-preventable disease (VPD) reports per year in Mexico during 2020-2024 and percentage of change.

## Abbreviations

The following abbreviations are used in this manuscript:

SINAVE	National Epidemiological Surveillance System
OECD	Organization for Economic Co-operation and Development
VPDs	vaccine-preventable diseases
NPIs	Non-pharmacological interventions
WEB	Weekly Epidemiological Bulletin
MoH	Mexican Ministry of Health
