# Supplementary material for: Impact of the COVID-19 Pandemic on Vaccine-Preventable Diseases in Mexico: A Time Series Analysis (2014–2024)
Source: Epidemiologia (Basel). 2026 Feb 11;7(1):26. doi: 10.3390/epidemiologia7010026 (PMC12921749; doi:10.3390/epidemiologia7010026)
Supplement: Supplementary file 1 [file epidemiologia-07-00026-s001.zip › epidemiologia-4078686-supplementary.pdf]

Supp. Table 1. Weekly median of vaccine-preventable disease (VPD) reports per year in Mexico during 2020-2024 and percentage of change.

Disease	Weekly median cases (2014-2019)	Weekly median cases (2020)	% change (2020)	Weekly median cases (2021)	% change (2021)	Weekly median cases (2022)	% change (2022)	Weekly median cases (2023)	% change (2023)	Weekly median cases (2024)	% change (2024)
Rotavirus, median (p25- p75)	21 (15 to 37)	4+ (3 to 8)	-81.0%	11+ (8 to 17)	-47.6%	12+ (7 to 22)	-45.2%	13* (9 to 29)	-38.1%	16+ 9 to 30)	-26.2%
Meningeal tuberculosis, median (p25-p75)	4 (3 to 7)	4 (3 to 6)	0.0%	9+ (6 to 11)	112.5%	11* (8 to 13)	175.0%	10** (8 to 13)	150.0%	11+ (6 to 13)	175.0%
Pulmonary tuberculosis, median (p25-p75)	285 (255 to 320)	419+ (268 to 548)	46.8%	333+ (267 to 358)	16.8%	399+ (327 to 435)	40.0%	409+ (357 to 444)	43.3%	418** (389 to 448)	46.7%
Chickenpox, median (p25-p75)	2,767 (1,909 to 2,767)	238+ (206 to 1,303)	-91.4%	289+ (272 to 339)	-89.6%	693+ (482 to 815)	-75.0%	774+ (644 to 940)	-72.0%	1,030+ (831 to 1,238)	-62.8%
Mumps, median (p25- p75)	86 (68 to 133)	47+ (36 to 93)	-45.0%	42+ (38 to 53)	-50.9%	49+ (42 to 62)	-42.7%	57+ (49 to 65)	-33.3%	73 (63 to 80)	-14.6%
Hepatitis A, median (p25-p75)	155 (120 to 199)	45+ (35 to 81)	-71.0%	9+ (30 to 53)	-75.2%	72+ (47 to 92)	-53.9%	129+ (104 to 154).	-17.1%	166 (125 to 207)	7.1%
Hepatitis B, median (p25-p75)	13 (10 to 16)	6+ (3 to 8)	-53.8%	8+ (6 to 11)	-38.5%	11 (8 to 16)	-19.2%	16** (13 to 18)	19.2%	16+ (12 to 19)	23.1%
HPV, median (p25-p75)	414 (343 to 493)	107+ (86 to 166)	-74.3%	190+ (156 to 219)	-51.4%	263+ (211 to 293)	-36.5%	257+ (223 to 284)	-38.0%	352+ (269 to 391)	-15.1%
Mild to moderate cervical dysplasia, median (p25-p75)	643 (557 to 725)	249+ (164 to 315)	-61.3%	515+ (325 to 588)	-19.9%	583** (508 to 644)	-9.3%	630 (550 to 694)	-1.9%	708+ (269 to 772)	10.2%
Severe cervical dysplasia and *in situ* cervical cancer, median (p25-p75)	77 (62 to 90)	37+ (26 to 48)	-51.9%	70 (49 to 92)	-9.7%	87** (74 to 108)	13.0%	97+ (82 to 103)	25.3%	89+ (75 to 101)	15.6%

Mann-Whitney U test compared to the endemic channel (2014-2019 period), *p<0.005, **p<0.0005,+p<0.00001.
